# Severity of Ionized Hypercalcemia and Hypocalcemia Is Associated With Etiology in Dogs and Cats

**DOI:** 10.3389/fvets.2019.00276

**Published:** 2019-08-22

**Authors:** Michelle Coady, Daniel J. Fletcher, Robert Goggs

**Affiliations:** Department of Clinical Sciences, Cornell University College of Veterinary Medicine, Ithaca, NY, United States

**Keywords:** malignancy, hyperparathyroidism, hypervitaminosis D, critical illness, kidney injury, hypoparathyroidism, eclampsia

## Abstract

**Background:** Calcium disorders are common in small animals, but few studies have investigated the etiology of ionized hypercalcemia and hypocalcemia in large populations. This study aimed to determine the incidence of ionized calcium disorders in dogs and cats treated at a tertiary referral clinic and to describe the associated diseases.

**Methods:** An electronic database of electrolyte analyses conducted at the Cornell University Hospital for Animals from 2007 to 2017 was searched. Dogs and cats with ionized hypercalcemia or hypocalcemia were identified based on institution reference intervals. Duplicate case entries were removed. Medical records were reviewed to identify the cause of the calcium abnormality. Chi-squared analysis with Bonferroni adjustment was performed to compare frequencies of disease processes between mild and moderate-severe disturbances.

**Results:** The database included 15,277 dogs and 3,715 cats. Hypercalcemia was identified in 1,641 dogs and 119 cats. The incidence of canine and feline hypercalcemia was 10.7 and 3.2%, respectively. Hypocalcemia was identified in 1,467 dogs and 450 cats. The incidence of canine and feline hypocalcemia was 9.6% and 12.1%, respectively. The most common pathologic causes of hypercalcemia in dogs were malignancy-associated (12.9%), parathyroid-dependent (4.6%) and hypoadrenocorticism (1.7%). In cats, malignancy-associated hypercalcemia (22.7%), kidney injury (13.4%) and idiopathic hypercalcemia (12.6%) were most common. Dogs presenting with moderate-severe hypercalcemia vs. mild hypercalcemia were significantly more likely to have hyperparathyroidism, malignancy-associated hypercalcemia or hypervitaminosis D, whereas cats were significantly more likely to have malignancy-associated hypercalcemia or idiopathic hypercalcemia. The most common pathologic causes of hypocalcemia in dogs were critical illness (17.4%), kidney injury (10.4%) and toxicity (7.5%). In cats, kidney injury (21.6%), urethral obstruction (15.1%), and critical illness (14.7%) were most frequent. Dogs presenting with moderate-severe hypocalcemia were significantly more likely to have hypoparathyroidism, kidney injury, eclampsia or critical illness, whereas cats were significantly more likely to have kidney injury, soft tissue trauma or urethral obstruction.

**Conclusions:** Mild calcium disturbances are most commonly associated with non-pathologic or transient conditions. Malignancy-associated hypercalcemia is the most common cause of ionized hypercalcemia in dogs and cats. Critical illness and kidney injury are frequent causes of ionized hypocalcemia in both species.

## Introduction

Hypercalcemia and hypocalcemia are commonly encountered in veterinary medicine with potentially life-threatening consequences. Calcium is essential for many cellular processes, including neuromuscular transmission, enzymatic reactions, blood clotting, vasomotor tone, and bone metabolism. Ionized calcium is the biologically active form and blood ionized calcium concentrations are tightly regulated through the concerted actions of parathyroid hormone (PTH), 1,25-dihydroxyvitamin D3 (calcitriol) and calcitonin. Parathyroid chief cells secrete PTH, which increases plasma calcium by mobilizing bone stores, increasing renal tubular calcium reabsorption, and increasing calcitriol synthesis. Calcitriol increases intestinal calcium uptake and enhances bone and renal calcium reabsorption. Hypocalcemia markedly increases PTH secretion with consequent increases in calcitriol concentrations that in turn inhibit PTH synthesis. Hypercalcemia suppresses PTH synthesis and stimulates calcitonin secretion thereby inhibiting osteoclastic bone resorption. Failure or disruption of these regulatory mechanisms results in hypercalcemia or hypocalcemia with accompanying clinical signs (1, 2).

Hypercalcemia may lead to vomiting, depression, weakness, muscle twitching, cardiac arrhythmias, and seizures. Hypocalcemia may cause muscle tremors, facial rubbing, muscle cramping, stiff gait, seizures, restlessness, aggression, hypersensitivity, and disorientation. Hypocalcemia also contributes to sepsis-associated myocardial dysfunction and to the development of ventricular arrhythmias and refractory hypotension. Severe ionized hypocalcemia may result in coagulation abnormalities (2–4). Even in the absence of clinical signs, abnormalities in calcium concentrations may provide diagnostic clues to underlying disease.

The causes of calcium disorders are well-described (1), but the incidence of these disorders in large populations is undefined and similarly, there is limited information regarding the relative frequencies of the underlying disorders. Previous retrospective studies of small patient populations suggest that malignancy is the most common cause of hypercalcemia in dogs with primary hyperparathyroidism, kidney disease and hypoadrenocorticism also prominent (5–7). There is little consistency among reports regarding common causes of hypercalcemia in cats, but idiopathic hypercalcemia, neoplasia, acute kidney injury (AKI) and urolithiasis are reported (8, 9). Commonly reported causes of hypocalcemia include hypoparathyroidism, chronic kidney disease (CKD), acute pancreatitis and eclampsia. It should be noted that much of the calcium disorder literature discusses only total calcium concentrations that are known to be unreliable indicators of the concentrations of biologically active ionized calcium (10, 11). The severity of the calcium disorder is associated with mortality in both dogs and cats (12, 13). Ionized calcium concentrations have non-linear U-shaped associations with case fatality rates in dogs and cats, wherein concentrations clustered around the RI midpoint had the lowest case fatality rates, while progressively abnormal concentrations were associated with proportionately increased risk of non-survival (12, 13).

The present study aimed to: determine the incidence of ionized calcium disorders in dogs and cats presenting to a tertiary referral facility; determine the frequency of the disease processes associated with ionized calcium disorders in these patients; evaluate the association between calcium disorder severity and the causal disease process. It was hypothesized that mild disturbances in calcium are associated with non-pathologic, transient or inconsequential causes, whereas moderate or severe disturbances are associated with pathologic causes such as paraneoplastic hypercalcemia and primary hyperparathyroidism for hypercalcemia, and eclampsia and primary hypoparathyroidism for hypocalcemia.

## Materials and Methods

### Electrolyte Analyses

Blood ionized calcium concentration measurements were conducted using a point-of-care analyzer (RapidPoint 405, Siemens, Malvern, PA, USA) equipped with ion-selective electrodes using blood samples collected into 1 mL syringes heparinized with dry balanced lithium/zinc heparin (Westmed Inc., Tucson, AZ, USA). Samples were run immediately following collection. The sampling device used remained constant throughout the study period. The analyzer performs an automatic quality control (QC) analysis on a schedule without operator intervention. An onboard QC cartridge contains three levels of QC material to monitor system performance and provides target ranges for each QC material level. During automatic QC analysis, the system compares the results to the ranges for each parameter and identifies results that are out of range. Parameters that fail QC are turned off. Repeat QC analysis (with within range values) is required to turn on failed parameters. At our institution the automatic QC is set to run levels one and three daily at 6 a.m. and levels one and two daily at 10 p.m. Local reference intervals (RIs) for this analyzer were previously generated (2007) from healthy animals (20 dogs and 20 cats) that were not part of the study population. Those animals were considered healthy on the basis of history, physical examination, and the results of complete blood count and serum chemistry profiles. The relevant RI for ionized calcium for dogs was 1.18–1.37 mmol/L and for cats was 1.07–1.47 mmol/L.

### Case Selection and Database Compilation

An electronic database of blood gas and electrolyte analyses conducted in the emergency room or intensive care unit at the Cornell University Hospital for Animals between 05/31/2007 and 01/03/2017 was searched for results from dogs and cats. Some data from this database have been previously reported (12–15). The database was visually inspected and manually curated to remove samples from species other than dogs and cats, samples with missing, erroneous or untraceable case numbers, analyses from sample types other than blood (e.g., abdominal fluid) and analyses with missing data. Institution electronic medical record (EMR) systems were searched for data on patient signalment, presenting complaint, final diagnosis, outcome, and hospitalization dates to create databases containing electrolyte data, point-of-care analyses, biochemistry analyses, and case demographics. A custom application (Visual Basic, Microsoft Visual Studio for Windows, Microsoft, Redmond, WA, USA) was written to search each database via the unique patient identifier and create a final composite database combining data from all of the separate databases corresponding to the time and date stamp from the electrolyte analyses. In patients for which multiple analyses were identified, only the first recorded measurement was used. The final database was then manually checked for accuracy by cross-referencing the database entries with the parent data sources for a randomized selection of cases, spanning the entire range of case numbers, and representing 0.1% of the total case entries.

From the parent database, cases with hypercalcemia and hypocalcemia were segregated for further analyses. The patients' EMR were manually reviewed to verify the accuracy of the final diagnosis and identify the etiology of the calcium abnormality. All cases were classified by the same individual (MC), allowing for consistency throughout the process. Detailed EMR review was not conducted where the final diagnosis (as adjudicated by the original attending clinician) indicated in the EMR was consistent with a known cause of a calcium disturbance. Detailed EMR review was conducted when the final diagnosis was not indicated, was open, or was not consistent with a known etiology of calcium disturbance. In these instances, one individual (MC) reviewed the EMR thoroughly by reading available records including medical history, physical examination findings, daily structured assessments, through independent assessment of laboratory and pathology data, review of treatments performed and analysis of client and referring veterinarian communications. These case reviews were continued until it was apparent how the etiology of the calcium disturbance should be classified. In cases where the situation remained unclear discussions between authors (MC/RG) occurred to determine the best way to classify the case.

Each entry was classified into a previously reported cause of hypercalcemia or hypocalcemia per Schenck et al. (1). Additional diseases previously associated with hypocalcemia were also considered, including critical illness, including sepsis and systemic inflammatory response syndrome, diabetes mellitus, including diabetic ketoacidosis (DKA) (2), and feline urethral obstruction (1). In the present study, critical illness was defined as any potentially life-threatening multisystem disorder that, in the absence of medical intervention, would be expected to result in mortality or significant morbidity (16, 17). Primarily this category included patients with evidence of systemic inflammatory response syndrome and sepsis. Patients with acute kidney injury, pancreatitis, toxicity or trauma were classified separately.

Non-pathologic and transient or inconsequential causes of hypercalcemia were combined into a single category. Patients were classified in this category only if no other cause of hypercalcemia was identifiable, if the hypercalcemia resolved on subsequent electrolyte analysis, or by the time of follow-up re-evaluation at the hospital. Animals under 1 year of age were also categorized into this group. This category therefore encompasses such instances as young growing animals (increased bone turnover), spurious results, hemoconcentration, and hyperproteinemia.

Cases of hypercalcemia or hypocalcemia were classified as “undetermined” if diagnostic tests were not performed, if follow-up was performed at the primary care veterinarian, or if the patient died or was euthanized prior to diagnosis. For example, a dog with lymphocytosis and hypercalcemia where the calcium disorder was suspected to be paraneoplastic but follow-up diagnostic testing was performed only by the primary care veterinarian and not recorded in the EMR would have been classified as “undetermined.” Cases where the EMR indicated a suspected cause of the calcium disturbance that could not be otherwise categorized were classified as “other.” When two potential causes of the calcium disorder were applicable to one patient, the disease process deemed most likely to be the cause following extensive EMR review was selected. Hypercalcemia entries were classified as malignancy-associated if the final diagnosis was cancer, if the patient's blood PTH-related peptide concentration was increased, or if the patient had a documented or suspected cancer known to be associated with hypercalcemia in the absence of an alternative cause.

Once all calcium disorders had been categorized based on cause, they were labeled as mild or moderate-severe. No definitions for mild or moderate-severe calcium disorders were identified in the veterinary literature and hence we developed a categorization that seemed reasonable based on our reference ranges and on the data set. Mild hypercalcemia was defined as an ionized calcium >1.37 mmol/L but <1.5 mmol/L in dogs and >1.47 mmol/L but <1.6 mmol/L in cats. Moderate-severe hypercalcemia was defined as an ionized calcium ≥1.5 mmol/L in dogs and ≥1.6 mmol/L in cats. Mild ionized hypocalcemia was defined as an ionized calcium ≤ 1.17 mmol/L but ≥1.00 mmol/L in dogs and ≤ 1.06 mmol/L but ≥1.00 mmol/L in cats. Moderate-severe hypocalcemia was defined as an ionized calcium <1.00 mmol/L in both dogs and cats.

### Statistical Analysis

Statistical analysis was performed using commercially available software (SPSS Statistics 23, IBM, Armonk, NY and Prism 7.0e, GraphPad, La Jolla, CA). Chi-squared analysis was used to compare the frequencies of each disease classification in patients with mild ionized calcium disturbances compared to those in patients with moderate-severe ionized calcium disturbances. Fisher's exact tests were used to compare frequencies of specific categories of underlying disorder between dogs and cats for both hypercalcemia (11 comparisons), and hypocalcemia (12 comparisons). For all analyses, *post-hoc* Bonferroni adjustments were applied to account for multiple comparisons. Alpha was set at 0.05.

## Results

### Incidence of Calcium Disturbances in Dogs

For dogs, 44,366 records were identified across the 9.5-year period. After removal of subsequent or follow-up analyses, the initial analyses from 15,277 individuals remained. Of these, 1,643 electrolyte profiles documented hypercalcemia. After removal of two profiles from dogs that were dead-on-arrival there were 1,641 profiles with hypercalcemia, corresponding to an incidence of canine hypercalcemia of 10.7% (1,641/15,277). Hypocalcemia was documented in 1,468 profiles. After removal of one profile from a miscoded Fennec fox there were 1,467 profiles with hypocalcemia, corresponding to an incidence of canine hypocalcemia of 9.6% (1,467/15,277).

### Incidence of Calcium Disturbances in Cats

For cats, 9,992 records were identified across the 9.5-year period. After removal of subsequent or follow-up analyses, the initial analyses from 3,715 individuals remained. Of these, 119 electrolyte profiles documented hypercalcemia, corresponding to an incidence of feline hypercalcemia of 3.2% (119/3,715). Hypocalcemia was documented in 450 profiles, corresponding to an incidence of feline hypocalcemia of 12.1% (450/3,715).

### Hypercalcemia in Dogs

Causes of hypercalcemia in dogs are summarized in Table 1. Non-pathologic, transient or inconsequential causes were most commonly identified, accounting for 70.6% (1,158/1,641) of cases. Malignancy-associated hypercalcemia occurred in 12.9% (212/1,641) of cases. Of the malignancy-associated cases, 37.7% (80/212) were associated with lymphoma, 22.2% (47/212) with a malignancy of unknown type, 11.8% (25/212) with anal sac apocrine gland carcinoma, 9.9% (21/212) with other carcinomas, 7.1% (15/212) with bone neoplasia, 5.2% (11/212) with sarcoma, 3.3% (7/212) with multiple myeloma, 1.9% (4/212) with leukemia and 0.9% (2/212) with thymoma. Parathyroid-dependent causes of hypercalcemia occurred in 4.4% (73/1,641) of cases, of which 46.6% (34/73) were due to parathyroid adenoma, 11.0% (8/73) were due to parathyroid hyperplasia, and 9.6% (7/73) were due to parathyroid carcinoma. The remaining 24 cases did not have available histopathology. Hypoadrenocorticism accounted for 1.7% (28/1,641) of cases. In addition, there were 19 cases of kidney injury (15 diagnosed as CKD and 4 as AKI), 10 cases of non-malignant skeletal lesions (eight cases of hypertrophic osteodystrophy and two cases of osteomyelitis), five cases of hypervitaminosis D and four cases of granulomatous disease (one case of systemic mycosis, one case of pulmonary blastomycosis, and one cases of panniculitis). Only three cases of canine hypercalcemia were classified as “other,” including one dog with moderate-severe hypercalcemia that was suspected to be a side-effect of lactulose administration, one dog with mild hypercalcemia suspected to be secondary to hydrochlorothiazide administration, and one dog with mild hypercalcemia secondary to over-supplementation of intravenous calcium for treatment of eclampsia. Overall, 7.9% (129/1,641) of cases were of undetermined cause. Chi-squared analysis indicated there were significantly more dogs with non-pathologic, transient, or inconsequential causes of hypercalcemia in cases where the ionized calcium concentrations were classified as mild compared to when concentrations were moderate-severe. In contrast, there were significantly more dogs with parathyroid-hormone dependent hypercalcemia, humoral hypercalcemia of malignancy and hypervitaminosis D in cases where the ionized calcium concentrations were classified as moderate-severe compared to when they were classified as mild. There were also significantly more cases of hypercalcemia classified as unknown cause in dogs with moderate-severe hypercalcemia.

**Table 1:** Conditions associated with hypercalcemia.

**Cause**	**Dogs**			**Cats**			***P***	***P*_**corr**_**
	**Mild *n* (%)**	**Moderate-severe *n* (%)**	**All *n* (%)**	**Mild *n* (%)**	**Moderate-severe *n* (%)**	**All *n* (%)**	**All dogs vs. all cats**	**Bonferroni corrected**
Non-pathologic, transient or inconsequential	1,126^a^ (81.1)	32^b^ (12.6)	1,158 (70.6)	33^a^ (40.2)	1^b^ (2.7)	34 (28.6)	<0.0001	**<0.001**
Hypoadrenocorticism	23^a^ (1.7)	5^a^ (2.0)	28 (1.7)	0 (0.0)	0 (0.0)	0 (0.0)	0.2544	>1.000
Parathyroid dependent	22^a^ (1.6)	51^b^ (20.2)	73 (4.6)	3^a^ (3.7)	1^a^ (2.7)	4 (3.4)	0.8155	>1.000
Malignancy associated	93^a^ (6.7)	119^b^ (47)	212 (12.9)	14^a^ (17.1)	13^b^ (35.1)	27 (22.7)	0.0051	0.0561
Idiopathic hypercalcemia	0 (0.0)	0 (0.0)	0 (0.0)	6^a^ (7.3)	9^b^ (24.3)	15 (12.6)	<0.0001	**<0.001**
Kidney Injury	15^a^ (1.1)	4^a^ (1.6)	19 (1.2)	13^a^ (15.9)	3^a^ (8.1)	16 (13.4)	<0.0001	**<0.001**
Hypervitaminosis D	2^a^ (0.1)	3^b^ (1.2)	5 (0.3)	0 (0.0)	0 (0.0)	0 (0.0)	1.000	>1.000
Granulomatous disease	3^a^ (0.2)	1^a^ (0.4)	4 (0.2)	0 (0.0)	0 (0.0)	0 (0.0)	1.000	>1.000
Skeletal lesions (non-malignant)	7^a^ (0.5)	3^a^ (1.2)	10 (0.6)	0 (0.0)	0 (0.0)	0 (0.0)	1.000	>1.000
Other	2^a^ (0.1)	1^a^ (0.4)	3 (0.2)	9^a^ (11.0)	3^a^ (8.1)	12 (10.1)	<0.0001	**<0.001**
Undetermined	95^a^ (6.8)	34^b^ (13.4)	129 (7.9)	4^a^ (4.9)	7^b^ (19.0)	11 (9.2)	0.5973	>1.000
All causes	1,388 (84.6)	253 (15.4)	1,641 (100)	82 (68.9)	37 (31.1)	119 (100)		

*Mild hypercalcemia was defined as an ionized calcium >1.37 mmol/L but <1.5 mmol/L in dogs and >1.47 mmol/L but <1.6 mmol/L in cats. Moderate-severe hypercalcemia was defined as an ionized calcium ≥1.5 mmol/L in dogs and ≥1.6 mmol/L in cats. Severity classifications sharing a superscript letter denote proportions that do not differ significantly from each other at the 0.05 level. after Bonferroni correction for multiple comparisons. P_corr_ denotes values corrected for multiple comparisons (n = 11) using the Bonferroni method. The P-values listed in bold type are statistically significant at P < 0.05 after correction for multiple comparisons*.

### Hypercalcemia in Cats

Causes of hypercalcemia in cats are reported in Table 1. Non-pathologic, transient or inconsequential, causes of hypercalcemia accounted for 28.6% (34/119) of cases. Hypercalcemia of malignancy accounted for 22.7% (27/119) of cases, with 11 cases of lymphoma, 8 cases of carcinoma, and 8 cases of a malignancy of unknown type. Kidney injury was the cause in 13.4% (16/119) of cases, 11 of which had CKD and 5 had AKI. Idiopathic hypercalcemia was observed in 12.6% (15/119) of cases. Only four cases of parathyroid-dependent hypercalcemia were identified in cats. Of these one was due to a parathyroid carcinoma, while the remaining three did not have available histopathology. Hypercalcemia was classified as “other” in 12 cats, including 2 cats who were over-supplemented with intravenous calcium, 2 cats with concurrent urolithiasis, and 8 cats whose hypercalcemia was suspected to be secondary to lactulose administration. The cause of hypercalcemia could not be determined in 11 cases. As was the case for dogs, there were significantly more cats with non-pathologic causes of hypercalcemia where the ionized calcium concentrations were mildly increased compared to when the concentrations were moderately-to-severely increased. Ionized hypercalcemia was significantly more likely to be moderate-severe than mild in cats with idiopathic hypercalcemia or hypercalcemia of malignancy. Again, like dogs, there were significantly more cases of hypercalcemia of undetermined cause in cats with moderate-severe hypercalcemia.

### Hypocalcemia in Dogs

Causes of hypocalcemia in dogs are summarized in Table 2. Non-pathologic causes accounted for 44% (646/1,467) of cases. Critical illness accounted for 17.4% (255/1,467) of cases, followed by kidney injury accounting for 10.4% (152/1,467) of cases. Toxicity was the cause in 7.5% (110/1,467) cases of hypocalcemia, with 62.7% (69/110) of those cases due to suspected citrate toxicity from blood transfusion, 36.4% (40/110) cases due to furosemide administration, and one case due to ethylene glycol intoxication. Nutritional or gastrointestinal causes were responsible for 6.3% (93/1,467) cases, all of which were due to malabsorptive disorders. Diabetes mellitus was responsible for 4.4% (64/1,467) of cases. Of these, 53% (34/64) had diabetic ketoacidosis and 21% (14/64) had concurrent acute pancreatitis. Acute pancreatitis alone accounted for 3.5% (51/1,467) of cases, while soft tissue injury accounted for 1.0% (14/1,467) of cases. Of the 11 parathyroid-related cases, 6 were postoperative parathyroidectomy patients and 5 were diagnosed with primary hypoparathyroidism. No cause could be determined for 2.2% (33/1,467) of cases. There were significantly more dogs with non-pathologic hypocalcemia where the ionized calcium concentrations were mildly decreased compared to when the concentrations were moderately-to-severely decreased. In contrast, there were significantly more dogs with parathyroid-hormone dependent hypocalcemia, kidney injury, eclampsia, and critical illness in cases where ionized calcium concentrations were moderately-to-severely decreased compared to when they were only mildly decreased. Again, significantly more cases of hypocalcemia were classified as undetermined in dogs with moderate-severe hypocalcemia.

**Table 2:** Conditions associated with hypocalcemia.

**Cause**	**Dogs**			**Cats**			***P***	***P*_**corr**_**
	**Mild *n* (%)**	**Moderate-severe *n* (%)**	**All *n* (%)**	**Mild *n* (%)**	**Moderate-severe *n* (%)**	**All *n* (%)**	**All dogs vs. all cats**	**Bonferroni corrected**
Non-pathologic, transient or inconsequential	627^a^ (49.8)	19^b^ (9.2)	646 (44.0)	87^a^ (40.1)	22^b^ (9.4)	109 (24.3)	<0.0001	**<0.001**
Parathyroid related	7^a^ (0.6)	4^b^ (1.9)	11 (0.7)	2^a^ (0.9)	0^a^ (0.0)	2 (0.4)	0.7443	>1.000
Kidney injury	99^a^ (7.9)	53^b^ (25.6)	152 (10.4)	36^a^ (16.6)	61^b^ (26.2)	97 (21.6)	<0.0001	**<0.001**
Acute pancreatitis	43^a^ (3.4)	8^a^ (3.9)	51 (3.5)	5^a^ (2.3)	6^a^ (2.6)	11 (2.4)	0.3602	>1.000
Nutritional or gastrointestinal	77^a^ (6.1)	16^a^ (7.7)	93 (6.3)	6^a^ (2.8)	5^a^ (2.1)	11 (2.4)	0.0008	**0.0096**
Eclampsia	23^a^ (1.8)	15^b^ (7.2)	38 (2.6)	0 (0.0)	0 (0.0)	0 (0.0)	<0.0001	**<0.001**
Toxicity	95^a^ (7.5)	15^a^ (7.2)	110 (7.5)	20^a^ (9.2)	18^a^ (7.7)	38 (8.4)	0.5446	>1.000
Critical illness	206^a^ (16.3)	49^b^ (23.7)	255 (17.4)	25^a^ (11.5)	41^a^ (17.6)	66 (14.7)	0.1940	>1.000
Diabetes mellitus	54^a^ (4.3)	10^a^ (4.8)	64 (4.4)	15^a^ (6.9)	14^a^ (6.0)	29 (6.4)	0.0791	0.9492
Soft tissue trauma	14^a^ (1.1)	0^a^ (0.0)	14 (1.0)	1^a^ (0.5)	7^b^ (3.0)	8 (1.8)	0.2016	>1.000
Urethral obstruction	0 (0.0)	0 (0.0)	0 (0.0)	19^a^ (8.8)	49^b^ (21.0)	68 (15.1)	<0.0001	**<0.001**
Undetermined	15^a^ (1.2)	18^b^ (8.7)	33 (2.2)	1^a^ (0.5)	10^b^ (4.3)	11 (2.4)	0.8571	>1.000
All causes	1,260 (85.9)	207 (14.1)	1,467 (100)	217 (48.2)	233 (51.8)	450 (100)		

*Mild hypocalcemia was defined as an ionized calcium ≥1.00 mmol/L but ≤ 1.17 mmol/L in dogs and ≥1.00 mmol/L but ≤ 1.06 mmol/L in cats. Moderate-severe hypocalcemia was defined as an ionized calcium <1.00 mmol/L in both dogs and cats. Severity classifications sharing a superscript letter denote proportions that do not differ significantly from each other at the 0.05 level after Bonferroni correction for multiple comparisons. P_corr_ denotes values corrected for multiple comparisons (n = 12) using the Bonferroni method. The P-values listed in bold type are statistically significant at P < 0.05 after correction for multiple comparisons*.

### Hypocalcemia in Cats

Causes of hypocalcemia in cats are summarized in Table 2. Non-pathologic, transient or inconsequential causes of hypocalcemia accounted for 24.2% (109/450) of cases. Kidney injury was the most common pathologic cause of hypocalcemia, accounting for 21.6% (97/450) of cases, of which 62.9% (61/97) were diagnosed as AKI and 37.1% (36/97) were diagnosed as CKD. Urethral obstruction accounted for 15.1% (68/450) of cases, while 14.7% (66/450) were due to critical illness. Toxicities accounted for 8.4% (38/450) of cases, of which 71.1% (27/38) were due to suspected citrate toxicity from transfusion and 28.9% (11/38) were due to furosemide administration. Of the 6.4% (29/450) diabetic causes of hypocalcemia, 19 patients had diabetic ketoacidosis and 2 patients had concurrent acute pancreatitis. Acute pancreatitis alone was present in 11 patients, malabsorptive gastrointestinal disease was present in 11 cases and 8 patients had soft tissue injury. Both of the two parathyroid-related cases were postoperative parathyroidectomy patients. A cause could not be determined in 11 cases of hypocalcemia. There were significantly more cats with non-pathologic causes where the ionized calcium concentrations were mildly decreased compared to when concentrations were moderately-to-severely decreased. In contrast, there were significantly more cats with kidney injury, urethral obstruction, and soft tissue trauma in cases where the ionized concentrations were classified as moderately-to-severely decreased compared to when they were classified as mildly decreased. There were significantly more cases of hypocalcemia classified as undetermined in cats with moderate-severe hypocalcemia.

### Canine vs. Feline Comparisons

Comparisons of frequencies of causes of hypercalcemia between dogs and cats determined that non-pathologic causes of hypercalcemia were significantly more common in dogs than in cats, while kidney injury, idiopathic hypercalcemia, and “other” were significantly more common in cats than in dogs (all *P* < 0.001 after correction for multiple comparisons) (Table 1). Within causes of hypocalcemia, non-pathologic causes, nutritional or gastrointestinal causes and eclampsia were significantly more common in dogs than in cats, while urethral obstruction and kidney injury were significantly more common in cats than in dogs (all *P* < 0.01 after correction for multiple comparisons) (Table 2).

## Discussion

### Hypercalcemia

In dogs, the most common pathologic causes of ionized hypercalcemia were malignancy, primary hyperparathyroidism, hypoadrenocorticism and kidney injury. These findings are consistent with previous reports (5–7). In cats, malignancy, kidney injury, idiopathic hypercalcemia and hyperparathyroidism were most common. These findings are consistent with a study of 71 cats with total hypercalcemia (8). A recent review suggested that idiopathic hypercalcemia should be considered the predominant cause in cats (18). However, that article referred to unpublished data and prior reports available only in abstract form. Potential explanations for these disparities include inclusion of distinct patient populations, use of different analyzers or RIs, differences in the numbers of cats included, and dissimilarities in diagnostic investigation and disease classifications. Multicenter studies pooling data from multiple patient populations with consistent analyzer measurements and common definitions might help to address this issue. Idiopathic hypercalcemia is an elusive disease generally considered a diagnosis of exclusion. In the present study, cats with idiopathic hypercalcemia more frequently had moderate-severe hypercalcemia, suggesting that this differential should be prioritized in cats presenting with marked increases in ionized calcium.

In the present study, the degree of hypercalcemia was associated with the frequency of the underlying disorder. Dogs evaluated at our institution with moderate-severe hypercalcemia were significantly more likely to have hyperparathyroidism, malignancy or hypervitaminosis D than those with mild hypercalcemia. In contrast, cats with moderate-severe hypercalcemia at our institution were significantly more likely to have malignancy or idiopathic hypercalcemia than if the hypercalcemia was mild.

Malignancy was the most frequently identified pathologic cause of both canine and feline ionized hypercalcemia in the present study. Cancer may result in hypercalcemia through various mechanisms including PTHrp, cytokines, bone resorption due to hematologic malignancies present in marrow and tumor metastasis to bone with subsequent local bone resorption (1). Lymphoma, which can cause humoral hypercalcemia, was the most common malignancy identified in both species here, consistent with previous reports (5, 7). In the present study, it is possible that hypercalcemia was incorrectly attributed to malignancy in some cases. Patients were classified as malignancy-associated if they had a documented neoplastic process and increased calcium concentrations. While this strategy may have falsely increased the overall incidence of malignancy associated hypercalcemia it would not have affected comparisons between mild and moderate-severe groups or between species because the method was uniformly applied to all cases.

Primary hyperparathyroidism was a common diagnosis in both cats and dogs in the present study. Primary hyperparathyroidism is typically considered rare in cats compared to dogs, but the disorder occurred with comparable frequency in the present study. Histopathology was available for most canine cases that were predominantly due to parathyroid adenomas, a finding consistent with the literature (19). Only one cat had available histopathology, indicating parathyroid carcinoma. The similar incidence between species identified here may have resulted from an increased secondary and tertiary patient population at our institution that might bias toward unusual cases. Alternatively, our larger sample size may have enabled us to identify the true incidence of primary hyperparathyroidism in cats. Our findings will require replication in another population to confirm which of these is correct.

Kidney injury was a more frequent cause of ionized hypercalcemia in cats than in dogs. Most cases in both species were diagnosed as CKD rather than AKI. Hypercalcemia is reported to occur in ~30% of cats with CKD and ~10% of dogs with CKD (10, 11), through various mechanisms including renal hyperparathyroidism, decreased glomerular filtration, increased tubular reabsorption and decreased bone storage. For the CKD patients presented here, parathyroid concentrations were not available, so it is possible that renal hyperparathyroidism was present but undiagnosed. Hypercalcemia can itself contribute to development or propagation of kidney injury and hence we cannot exclude the possibility that the kidney injury identified as the cause in these patients was in fact secondary. Urolithiasis has been previously associated with ionized hypercalcemia in cats. Urolithiasis was present in 11 of 71 cats with hypercalcemia in one study (8). Hypercalciuria may predispose patients to form calcium-containing uroliths and hence it is possible that the cats with urolithiasis in this study had a separate undiagnosed cause of hypercalcemia. Lactulose administration was suspected to be the cause of hypercalcemia in 8/119 cats in this study. An experimental study in dogs identified that lactulose administration causes increased intestinal absorption of calcium and magnesium likely through alterations in intestinal pH (20), although to our knowledge a clinical association between lactulose administration and hypercalcemia has not been reported in human or veterinary medicine to date.

The association of hypervitaminosis D with moderate-severe hypercalcemia identified in this study is weak. The total number of cases of hypervitaminosis D in this study (*n* = 5) was small compared to malignancy (*n* = 212) or primary hyperparathyroidism (*n* = 73) for instance. As a result, the difference between the two categories of severity were statistically significantly different, but this was based on the difference of a single case between the categories. This apparent difference should be interpreted with caution and hypervitaminosis D remains an important differential diagnosis even in patients with mild hypercalcemia.

### Hypocalcemia

The most common pathologic causes of ionized hypocalcemia identified in both dogs and cats in the present report were critical illness and kidney injury, with urethral obstruction also common in cats. Dogs presenting with moderate-severe hypocalcemia were significantly more likely to have hypoparathyroidism, kidney injury, eclampsia or critical illness, whereas cats were significantly more likely to have kidney injury, soft tissue trauma, or urethral obstruction. Fortunately, many of these differentials can be ruled out based on history and physical exam alone. Many of the disorders resulting in hypocalcemia, such as kidney injury, diabetes mellitus, pancreatitis, and critical illness, are more challenging to separate given that many of these diseases may coexist. In the present study, attempts were made to prioritize the disease that was resulting in the patient's admission to the hospital, but it is possible that overlap in disease processes may have altered the results.

Ionized hypocalcemia is very common in critical illness, occurring in over half of critically-ill people (21, 22), 16–24% of critically-ill dogs (23–25) and 59–93% of cats with septic peritonitis (26, 27). The mechanisms by which critical illness and hypocalcemia are associated are poorly understood and are likely multifactorial. Alterations in parathyroid hormone, vitamin D deficiency, hypomagnesemia and tissue accumulation have all been proposed (3). Critically ill patients may also be predisposed to developing hypocalcemia due to concurrent disease processes or treatments, such as blood transfusions, aggressive intravenous fluid therapy, concurrent kidney injury or pancreatitis. In the present study, critical illness accounted for 17.4 and 14.7% of hypocalcemia cases in dogs and cats, respectively. Critical illness was more frequent in dogs with moderate-severe hypocalcemia, occurring in 23.7% of cases. In people, ionized hypocalcemia is associated with illness severity but is also an independent predictor of mortality (21). Ionized hypocalcemia is associated with mortality in both dogs and cats (12, 13) and with duration of ICU stay and length of hospitalization (23, 24, 27). Failure of ionized calcium concentrations to normalize during hospitalization is a negative prognostic indicator in cats (27).

Hypocalcemia associated with AKI was a common cause of hypocalcemia in both dogs and cats in this study. In AKI, hyperphosphatemia may occur secondary to decreased glomerular filtration rate, which in turn may result in hypocalcemia due to increased binding by phosphate (1). Hypocalcemia in urethral obstruction may be due to phosphate retention secondary to obstruction, PTH resistance, or acid-base alterations. Ionized hypocalcemia is reported in 75% of cats presenting with urethral obstruction (28). Mild ionized hypocalcemia is common in dogs and cats with DKA and may be due to osmotic diuresis, supplementation of bicarbonate or potassium phosphate, or concurrent pancreatitis or AKI. Diabetes mellitus was a frequent cause of hypocalcemia in the present study, occurring in 4.4% of hypocalcemic dogs and 6.4% of hypocalcemic cats. The majority of these patients had DKA. In a study of 127 dogs with DKA, 52% had ionized hypocalcemia, which was associated with non-survival (29). Ionized hypocalcemia is also frequently identified in cats with pancreatitis, and is considered a risk factor for mortality (30, 31). The hypocalcemia in pancreatitis is hypothesized to be due to increased calcitonin, calcium sequestration by peripancreatic fat and free fatty acids, and through hypomagnesemia induced PTH resistance (1). Pancreatitis was an uncommon cause of hypocalcemia in the present study and the frequency of pancreatitis did not vary with hypocalcemia severity. This finding is consistent with the variability in calcium concentrations reported for typical acute pancreatitis populations (30).

Eclampsia, or puerperal tetany, occurs in the periparturient period in dogs or cats due to depletion of ionized calcium that occurs with lactation (32). In this study, eclampsia more frequently caused severe hypocalcemia, but only in dogs. Toxicity was also an important cause of hypocalcemia in both dogs and cats in the present study and typically resulted from furosemide administration or citrate toxicity associated with blood transfusion. Furosemide is commonly prescribed for calciuresis in hypercalcemic patients, but hypocalcemia is more commonly a side-effect of loop diuretic usage in patients with congestive heart failure. In one study of 10 dogs receiving massive transfusion, all dogs experienced ionized hypocalcemia with two dogs experiencing ionized hypocalcemia <0.7 mmol/L (33).

There are limitations to the present study. In some cases, the cause of the calcium disturbance was not clearly stated in the EMR, which required categorizing the cause based on our comprehensive EMR review and considering any other diagnoses present. By necessity therefore subjective judgments were made about diagnoses that are prone to the biases inherent to retrospective studies. Similarly, in patients with comorbidities, the disease process deemed most likely to be the cause was recorded as the etiology. This may have led to misclassification. These judgments were made uniformly across all cases however, which may limit any impact on comparisons within the patient population. It is possible that some patients in this study had alternative causes of hypercalcemia or hypocalcemia that have not been previously reported. These could not have been identified based on retrospective record review. The measurements of ionized calcium were not corrected for pH, which may have altered the overall incidence of the calcium disturbances identified (34). Ionized calcium concentrations in this study were measured on heparinized whole blood samples and it is known that ionized calcium is less stable in heparinized blood compared to plasma. It is standard practice in our institution to measure electrolyte concentrations immediately post-collection which would have minimized any impact of sample type on measured concentration. The cause of the calcium disturbance was undetermined in many cases, particularly in patients with moderate-severe disorders. In many cases this indeterminate status related to the death or euthanasia of the patient prior to establishment of a definitive diagnosis, an outcome that may have been more common in patients with moderate-severe calcium disorders.

The present study used RIs generated in 2007 based on 20 dogs and 20 cats. In 2012, the American Society of Veterinary Clinical Pathology recommended that RIs be generated from populations containing a minimum of 40 animals (35). It is possible that a new RI generated from larger numbers of healthy animals might have led to differences in the percentages of patients in some groups in the present study. Recently, RIs for the RapidPoint 500 machine (the next generation of the analyzer used in the present study) have been published (36, 37). The canine ionized calcium RI published by Bachmann et al. (37) was 1.23–1.40 mmol/L based on 51 dogs. This is quite comparable to the interval used at our institution (1.18–1.37). Application of this published RI to our data would likely affect the distribution of cases across the three categories of hypercalcemia and of hypocalcemia, but it seems likely the broad conclusions would be similar. In the study by Bachmann et al. (36) a RI for ionized calcium in cats (*n* = 24) was not reported because their data were non-parametric. In that study, the median (min-max) values were 1.30 (1.18–1.35). This is a narrower range than that used in the present study but has a comparable mid-point the interval used at our institution. Similar to the situation in dogs, it is probable that applying the range of values from Bachmann et al. (36) would affect the distribution of cases across the three categories of hypercalcemia and of hypocalcemia, but would leave the broad conclusions from the present study unaltered. One finding in the present study that might be impacted through use of a narrower RI in cats is the difference in the percentage of mild hypocalcemia in cats compared to dogs (48.2 vs. 85.9%). This could be due to interspecies differences, but given the disparities in the RIs is more likely due to the wider RI in cats and the use of a fixed (>1.00 mmol/L) cutoff for defining mild hypocalcemia.

In summary, dogs presenting with moderate-severe hypocalcemia were significantly more likely to have hypoparathyroidism, kidney injury, eclampsia or critical illness, whereas cats were significantly more likely to have kidney injury, soft tissue trauma or urethral obstruction. Mild calcium disturbances are commonly associated with non-pathologic or transient conditions. Malignancy-associated hypercalcemia is the most common cause of ionized hypercalcemia in dogs and cats. Critical illness and kidney injury are frequent causes of ionized hypocalcemia in both species. In both dogs and cats with either hypercalcemia or hypocalcemia, mild disturbances were more likely to be non-pathologic. This may provide some justification to monitor and reconfirm mild calcium disturbances prior to pursuing further testing. In contrast, moderate-severe calcium disturbances warrant further investigation because of the likelihood of a pathologic cause in these patients.

## Data Availability

Anonymized datasets generated for this study are available upon reasonable request to the corresponding author.

## Ethics Statement

This study was exempt from ethics committee approval because it presents a retrospective analysis of electrolyte data collected as part of clinician-driven care provided to patients at the institution hospital. No client or patient identifying information is presented.

## Author Contributions

MC collected and analyzed data and wrote the manuscript. DF collected data and edited the manuscript. RG conceived the study, analyzed data, and edited the manuscript.

### Conflict of Interest Statement

The authors declare that the research was conducted in the absence of any commercial or financial relationships that could be construed as a potential conflict of interest.

## Abbreviations

AKI: acute kidney injury
CKD: chronic kidney disease
DKA: diabetic ketoacidosis
PTH: parathyroid hormone
PTHrp: parathyroid hormone related protein.
